# A survey of the sociodemographic and educational characteristics of oral health technicians in public primary health care teams in Minas Gerais, Brazil

**DOI:** 10.1186/1478-4491-11-67

**Published:** 2013-12-23

**Authors:** Mauro Henrique Nogueira Guimarães Abreu, Carla Aparecida Sanglard-Oliveira, Abdul Rahman Mustafá Jaruche, Juliana Vaz de Melo Mambrini, Marcos Azeredo Furquim Werneck, Simone Dutra Lucas

**Affiliations:** 1 Universidade Federal de Minas Gerais, Avenida Antônio Carlos, CEP: 31270-901, 6627 Belo Horizonte, Brazil; 2 René Rachou Research Center, FIOCRUZ, Avenida Augusto de Lima, CEP: 30190-002, 1715 Belo Horizonte, Brazil

**Keywords:** Dental auxiliaries, Dental hygienists, Oral health technician, Socioeconomic factors, Educational profile

## Abstract

**Background:**

To describe some sociodemographic and educational characteristics of oral health technicians (OHTs) in public primary health care teams in the state of Minas Gerais, Brazil.

**Methods:**

A cross-sectional descriptive study was performed based on the telephone survey of a representative sample comprising 231 individuals. A pre-tested instrument was used for the data collection, including questions on gender, age in years, years of work as an OHT, years since graduation as an OHT, formal schooling, individual income in a month, and participation in continuing educational programmes. The descriptive statistic was developed and the formation of clusters, by the agglomerative hierarchy technique based on the furthest neighbour, was based on the age, years of work as an OHT, time since graduation as an OHT, formal schooling, individual income in a month, and participation in continuing educational programmes.

**Results:**

Most interviewees (97.1%) were female. A monthly income of USD 300.00 to 600.00 was reported by 77.5% of the sample. Having educational qualifications in excess of their role was reported by approximately 20% of the participants. The median time since graduation was six years, and half of the sample had worked for four years as an OHT. Most interviewees (67.6%) reported having participated in professional continuing educational programmes. Two different clusters were identified based on the sociodemographic and educational characteristics of the sample.

**Conclusions:**

The Brazilian OHTs in public primary health care teams in the state of Minas Gerais are mostly female who have had little time since graduation, working experience, and formal schooling sufficient for professional practice.

## Background

The evaluation of the dental workforce globally is an important and actual issue. There are two kinds of auxiliary dental personnel in Brazil, oral health technicians (OHTs) and dental assistants. Within the context of health care work, the OHT contributes to integral patient care in both the private and public settings of the health care system by acting to support diagnosis, education on health, protection, prevention, recovery, rehabilitation, and health management. The OHT plays a crucial role in the promotion, prevention, and recovery of oral health at the individual and collective levels in the public and private settings. Dental assistants have less function in health care settings than OHTs, devoting more time to dental health promotion activities than to helping perform dental tasks with individual patients. Although accepted as an occupation since 1975, OHTs were only regulated in Brazil in 2008. According to this regulation patients cannot access OHTs directly for a diagnosis and treatment plan and for the few activities in which OHTs work directly with the oral cavity of the patient, the direct supervision of the dentist is mandatory. OHTs should have at least 11 years of formal education. Beyond clinical work, many community actions could be developed by these professionals [1,2]. Brazilian OHTs from Minas Gerais have worked more with preventive/collective activities than in individual clinical care [3].

In other countries [4,5], professions with job descriptions similar to that of the OHT have been assessed as to their sociodemographic and educational characteristics. Given the importance of human resources in the national health care system [6], such information is relevant for the planning of policies for health care human resources.

The present research study seeks to examine the sociodemographic and educational characteristics of a representative sample of OHTs in the state of Minas Gerais, Brazil. During the year when the present study was conducted, the state of Minas Gerais hosted approximately one-third of all OHTs in the Family Health Strategy (FHS) [7]. The latter is the country’s primary instrument for providing basic care to the population in Brazil [8]. Therefore, the present study is relevant for the Brazilian public health system (Unified Health System) [9], as more thorough knowledge of this human resource is crucial for the effective planning and management of primary health care. In addition, it might represent an effective contribution to the knowledge of personal and regional characteristics of OHTs, thus strengthening the theoretical-practical coherence of the work process. Besides, in Brazil, no such studies have been conducted with representative samples of OHTs. The availability of rigorous quantitative surveys of health care occupations could be useful in identifying appropriate solutions for human resources for health planning and management [10]. Health actions and health evaluation should consider the profile of health professions. Thus, the evaluation of personal characteristics of OHTs could be useful for a broader and better knowledge about their potential labour force in the FHS.

As a function of the new legislation for OHTs in Brazil and the relevance of such professionals for the rationale of the organization of the work process, especially within the context of the Unified Health System, the aim of the present study was to describe some sociodemographic and educational characteristics of Brazilian OHTs in public primary health care teams in the state of Minas Gerais.

## Methods

Minas Gerais state is the second most populous state in Brazil with 19,597,330 inhabitants in 2010, with a population growth of 9.51% in the last ten years.

Structured interviews were conducted by telephone with OHTs enrolled in the FHS of the state of Minas Gerais [11]. The OHTs’ names and workplaces were located through the website of the National Register of Health Institutions, which also supplied the number of dental teams that included an OHT registered with the FHS in the state of Minas Gerais for calculation of the sample size.

A questionnaire was elaborated based on a pre-existing instrument [12] that was adapted for the purpose of the present study and according to the attributes established by the law regulating the OHT profession in Brazil. The sociodemographic and educational variables assessed were the following: gender, age in years, years of work as an OHT, years since graduation as an OHT, formal schooling, individual income in a month, and participation in continuing educational programmes (yes/no). The instrument’s reliability was assessed by means of a test-retest conducted by telephone with 20 OHTs in the FHS of the state of Minas Gerais who were selected in a simple random manner. Agreement was estimated using the Cohen’s kappa statistic (categorical variables) and the intraclass correlation coefficient (quantitative variables) using the Statistical Package for the Social Sciences (SPSS for Windows, version 18.0; SPSS Inc, Chicago, IL, USA) software. Kappa coefficient values higher than 0.60 were considered to be adequate. Kappa values were lower than 0.60, that is, with low reliability, in only 5% of the questions, which were thus reformulated and tested anew with 12 additional randomly selected OHTs. The data were then reanalysed, and the results showed kappa values higher than 0.60 for all the questions. The intraclass correlation coefficient of the quantitative variables varied from 0.99 to 1, and no further measurements were therefore needed. Thus, the questionnaire was rated as appropriate for measuring the study variables.

The questionnaires were applied by telephone. The OHTs responded to the questionnaire at their workplace or another location on the date and time that they judged to be the most convenient. At the beginning of the interviews, the purpose of the study was explained to the participants, and the text of the free and informed consent form was read aloud by the interviewer.

The population (N = 545) was the same as the number of FHS dental teams that included an OHT in the state of Minas Gerais listed in the National Register of Health Institutions in May 2010. The latter is a national record of all health units linked to the Unified Health System [13]. The names of repeated OHTs and those who participated in the pilot study were excluded; therefore, the finite population of OHTs was 484.

The sample size was calculated based on a 50% expected frequency, 95% confidence interval, 5% precision, and finite population of OHTs equal to 484. Thus, the sample comprised 231 OHTs who were selected in a simple random manner.

From the 231 OHTs who composed the initial sample, 27 were lost for analysis (response rate = 88.3%) due to the following reasons: no longer working for the FHS (n = 12); refusal to participate in the study (n = 4); and impossibility to establish contact (n = 11). Therefore, the final sample comprised 204 OHTs, and the recalculated precision was 5.21%.

The data were analysed using the SPSS software for Windows, version 17.0. The descriptive statistics involved a proportion calculation, central tendency measures, and 95% confidence intervals. The assumptions of normality for numeric variables were evaluated with the Kolmogorov-Smirnov test. The clustering was based on age (in years), years of work as an OHT, years since graduation as an OHT, formal schooling (incomplete primary education; complete primary education; incomplete secondary education; complete secondary education; incomplete university level; complete university level), individual income in a month (categorized according to the Brazilian minimum wage), and participation in continuing educational programmes (the question was: ‘Do you attend any continuing educational programmes in the dental field?’ yes/no). The first three numeric variables were dichotomized regarding their median values. Formal schooling was dichotomized based on the minimum education needed to be an OHT (complete secondary education, that is, 11 years). Three types of clusters (of two to four clusters) were formed from the 204 OHTs in Minas Gerais. The choice of two clusters was due to a better understanding of the phenomenon (sociodemographic and educational characteristics). The multivariate agglomerative hierarchy technique based on the furthest neighbour was used for the cluster analysis (CA), which is an exploratory data analysis tool for organizing observed data (in our case, OHTs) into groups (clusters), based on combinations of independent variables (in our case, sociodemographic and educational characteristics of OHTs), and maximizes the similarity of cases within each cluster while maximizing the dissimilarity between groups. This multivariate analysis creates new groupings without any preconceived notion of what clusters may arise. This data reduction makes it easier for the management of subgroups. One of the difficulties of CA is to identify the number of clusters. In our study, as described previously, the choice of two clusters was due to a better understanding of the phenomenon. Clusters are also interpreted based solely on the variables included in them [14].

The study was submitted to and approved by the Ethics Committee for Human Research of the Federal University of Minas (Universidade Federal de Minas; protocol number 0027.0.203.000-10).

## Results

The overall description for each variable showed that regarding the sociodemographic variables, 97.1% of the OHTs were female (95% CI: 93.4 to 98.8%) and the same proportion graduated in OHT. The age range was 22 to 55 years, while 50% of the OHTs were 36 years old or younger. The monthly income of most OHTs (77.5%; 95% CI: 70.9 to 82.8%) varied from BRL 510.00 (USD 300.00) to BRL 1,020.00 (USD 600.00), whereas it was less than BRL 510.00 (USD 300.00) in less than 5% of the sample and higher than BRL 1,020.00 (USD 600.00) in 17.6% (95% CI: 12.8 to 23.7%). Regarding the educational level, 79.9% of the participants had up to 11 years of formal schooling. The time since graduation varied from one to 25 years, with a median of six years. A total of 67.6% of interviewees (95% CI: 60.7 to 73.9%) reported having participated in continuing educational programmes in dentistry while working as an OHT at the FHS. Their work experience as an OHT varied from zero to 20 years, while 50% of the interviewees had worked for four years as an OHT (Table 1). The sociodemographic and educational characteristics in each cluster are presented in Table 2.

**Table 1:** Descriptive analysis of sociodemographic variables of OHTs, Brazil, 2010

**Variables (n = 204)**	**% (95% CI)**
Female	97.1 (93.4 to 98.8)
Up to 11 years of formal schooling	79.9 (73.6 to 85.0)
Participation in continuing educational programmes	67.6 (60.7 to 73.9)
Individual income in a month	
Up to $300.00	4.9 (2.5 to 9.1)
$300.00 to $600.00	77.5 (71.0 to 82.9)
More than $600.00	17.6 (12.8 to 23.7)
Age in years (minimum; median; maximum)^a^	22; 36; 55
Years of work as an OHT (minimum; median; maximum)^a^	0; 4; 20
Years since graduation as an OHT (minimum; median; maximum)^a^	1; 6; 25

^a^No quantitative variable had a normal distribution (Kolmogorov-Smirnov tests; *P* <0.05). CI, confidence interval; OHT, oral health technician.

**Table 2:** Sociodemographic characteristics of OHTs from the two clusters, Brazil, 2010

**Variables**	**Cluster 1 (n = 159)**	**Cluster 2 (n = 35)**
	**% (95% CI)**	**% (95% CI)**
More than 11 years of formal schooling	14.5 (9.6 to 20.6)	45.7 (29.9 to 62.2)
Participation in continuing educational programmes	66.7 (59.1 to 73.7)	77.1 (61.2 to 88.8)
Individual income in a month		
Up to $300.00	6.3 (3.2 to 10.9)	0 (0 to 8.2)
$300.00 to $600.00	93.7 (89.1 to 96.8)	0 (0 to 8.2)
More than $600.00	0 (0 to 1.9)	100 (91.8 to 100)
More than 37 years old	30.2 (23.4 to 37.7)	88.6 (74.7 to 96.3)
More than four years of work as an OHT	34.6 (27.5 to 42.2)	65.7 (49.0 to 79.9)
More than six years since graduation as an OHT	37.7 (30.5 to 45.5)	91.4 (78.4 to 97.8)

CI, confidence interval; OHT, oral health technician.

## Discussion

Our results show that most of the interviewed OHTs were female (97.1%), which agrees with the findings of other studies conducted with health care professionals [6] and auxiliary oral health professionals [4,5]. There is a historical process of feminization of the oral health professions [15]. However, in the auxiliary oral health professions, we could not say that there is a process of feminization, since it has always been a feminine profession, almost everywhere. In 1910, Alfred Fones developed the first training programme for young women, whom he called ‘dental hygienists’ [16,17]. From that time onwards, the profession has included mainly females due to historical and cultural reasons relative to the work organization and the devaluation of the profession itself [15]. Despite this, there is a huge salaries variation in the public sector, since it depends on the local policies and career plans from the municipalities, and in our research, 82.4% of OHTs perceived less than the mean income of the personnel without higher education in public sector [18]. Two bills on wages of technical professions in health care are under evaluation by the Brazilian National Congress. The proposed wage for an OHT is less than a half of the proposal of the nursing technician. Thus, the monthly wage of an OHT provides further proof of the little value attributed to this profession [19,20].

The age profile of the study sample, which is associated with the years since graduation and work as an OHT, indicate a relatively young group of people with a potentially long future in the profession. In our study, there are a few OHTs (n = 3; 1.5%) who reported less than 11 years of formal schooling (data not shown). This happened probably because the legislation is recent and some OHTs may have been formed in the health service itself. The percentage of OHTs with incomplete or complete university level (more than 11 years of formal schooling) indicates the phenomenon of overeducation, that is, having educational qualifications in excess of their role [21]. Despite being formally prepared for professional practice, the educational qualifications in excess of their role might paradoxically suggest a possibility to change professions. The reasons for leaving the dental workforce may involve a complex net of reasons [22] and should be addressed by future research. On the other hand, we could not discard the possibility of this phenomenon which may represent opportunities for the further professionalization of this sector of the dental team.

The time since graduation and work experience as an OHT of the present sample correspond to the first decade of the 21st century. Indeed, most Brazilian OHTs graduated between 2004 and 2009, which is possibly due to the influence of the National Policy of Oral Health that was launched in 2004. The policy establishes the joint collaboration of the human resources at the FHS, and within this context, the OHTs play a key role in increasing the access to health care services through actions of prevention, promotion, and clinical assistance [2,23]. Different from other countries [2,24], the OHTs in Brazil are widely incorporated into the public sector, which might explain the findings.

The participation in continuing educational programmes show that the OHTs seek to remain up-to-date on the practice of their profession. The participation of hygienists in this type of training was also assessed in other countries [25,26].

The identification in the present study of two OHT clusters based on the assessed variables indicates some findings that might be useful in the planning of policies of human resources. Compared to cluster 2, cluster 1 was composed of younger OHTs with less time since graduation, working experience, income, and schooling. On the one hand, the characteristics of cluster 2 could show that this profession appears to allow for educational and income growth. It was expected that the OTHs with more time of working experience and age have more opportunities to participate in continuing educational programmes and to have higher salaries, particularly if they work in the public sector. In general, the Brazilian public sector provides career plans and/or permanent job opportunities. In consequence, more time of work means more advantages with respect to salaries and the opportunities to have continuing education. On the other hand, the presence of a group of individuals who could desire a shift to other professions requiring higher educational qualifications after a few years of practice might represent a cause for concern. This phenomenon might be explained by the low value attributed to and historical conflicts faced by this profession in Brazil [1,2]. Further research about turning over of this profession in the public sector is needed. The differences in formal educational between the clusters could not be explained by a change in entry bar. The Brazilian law maintained the old obligation of at least 11 years of formal educational for being an OHT. Although cluster 2 comprised a lower number of OHTs, one might infer that cluster 1 would exhibit characteristics similar to those of cluster 2 in the near future, unless the historical conflicts in the professionalization [2] are solved.

It is important to discuss the profile of OHTs in Brazil in relation to dental auxiliaries or mid-level providers in other countries. Brazilian law prohibits OHTs from exercising their activities in an autonomous fashion. Besides, dental corporative interests exert an influence over professionalization of OHTs, especially in discussions regarding the permissible activities of these professionals in the oral cavity of patients [2]. Empirical data shows that Brazilian OHTs from Minas Gerais have spent more time in community actions than in individual clinical care [3]. This is not the reality of dental auxiliaries in other countries [27-29] who have permission to perform clinical procedures even without the supervision of the dentist. They can even set up their own offices and exclusively offer dental hygiene services [2,27-29]. The socioeconomic and educational characteristics of Brazilian OHTs may be explained by their professional context.

It is worth noting that the data collected in the present study were provided by the OHTs’ self-reports. The diagnosis of public health issues might be established by quantitative and/or qualitative methods. The methodological option for a quantitative theoretical framework in the present study does not deny the urgent need for qualitative studies, which allow an assessment of the full complexity of the work process. Currently, as the organization of primary health care in Brazil is based on the FHS and person-centred assistance [26], the role of OHTs must be assessed and most likely reformulated. Other quantitative research could be developed using more sociodemographic and educational variables. Nevertheless, the quantitative methods are useful to approach wide-encompassing phenomena with probabilistic representativeness. The sampled OHTs were geographically distributed in a very similar way to the population of OHTs in Minas Gerais (data not shown). The distribution of our sample reaffirms the external validity of our study. Other surveys conducted at the national level are also important. Although the telephonic surveys might produce results that are different from those achieved by other methods, they are valid, and their viability is satisfactory, especially for the assessment of health care services. In the present study, the method used exhibited adequate reproducibility, and as mentioned above, it is considered to be valid by several studies in the field of public health and social sciences [10,30-33].

## Conclusions

The Brazilian OHTs included in the FHS in the state of Minas Gerais are mostly female who have had little time since graduation, working experience, and formal schooling sufficient for professional practice. There were two different clusters of OHTs with differences in sociodemographic and educational characteristics.

## Abbreviations

CA: Cluster analysis; CI: Confidence interval; FHS: Family health strategy; OHT: Oral health technician.

## Competing interests

The authors declare that they have no competing interests.

## Authors’ contributions

MHNGA conceived of the study and participated in its design and coordination. CASO performed the literature review and data collection and participated in the conception and design of this study. ARMJ performed the literature review and construction of the data bank. JVMM performed the literature review and the statistical analysis and interpretation. MAFW and SDL participated in the conception and design of the study. All authors helped to draft the manuscript and read and approved the final manuscript.
